# Repetitive transcranial magnetic stimulation as an augmentative strategy for treatment-resistant depression, a meta-analysis of randomized, double-blind and sham-controlled study

**DOI:** 10.1186/s12888-014-0342-4

**Published:** 2014-11-30

**Authors:** Bangshan Liu, Yan Zhang, Li Zhang, Lingjiang Li

**Affiliations:** Mental Health Institute of The Second Xiangya Hospital, National Technology Institute of Psychiatry,Key Laboratory of Psychiatry and Mental Health of Hunan Province, Central South University, Changsha, Hunan China; Shenzhen Kangning Hospital of Guangdong Province, Shenzhen, Guangdong China

**Keywords:** Transcranial magnetic stimulation, Depression, Treatment-resistant

## Abstract

**Background:**

Dozens of randomized controlled trials (RCTs) and meta-analyses have demonstrated the efficacy of repetitive transcranial magnetic stimulation (rTMS) for major depressive disorder (MDD) treatment, but there has not been a meta-analysis report which evaluates the efficacy and tolerability of rTMS used as an augmentative strategy for antidepressants in treatment-resistant depression (TRD) treatment. We thus conducted this meta-analysis, aimed at clarifying whether rTMS enhances the efficacy of TRD.

**Methods:**

We searched MEDLINE and Cochrane Central Register of Controlled Trials for RCTs for studying the efficacy of rTMS *versus* (*vs*) sham condition when combined with antidepressants in TRD treatment, and screened the references of the previous meta-analysis about the rTMS for MDD treatment. Response rates and NNT were chose as the primary outcomes, and remission rates, change from baseline of HAMD scores, dropouts were used as secondary outcomes. For dichotomous data, an intention-to-treat analysis principle was applied; for continuous data, we calculated the standard mean difference between groups with a random-effect model. Sensitivity analysis was done to explore the source of heterogeneity and the factors which potentially impact the efficacy.

**Results:**

Seven RCTs were finally included in the meta-analysis. The total sample size was 279, with 171 in the rTMS group and 108 in the sham group. The pooled response and remission rate for the rTMS and sham group was 46.6% and 22.1%, respectively; the pooled odds ratio (OR) was 5.12 [95% confidence interval (CI) 2.11-12.45, z = 3.60, p = 0.0003, and the associated number needed to treat (NNT) was 3.4. rTMS group achieved a significant reduction of HAMD score than the sham group, the pooled SMD of change from baseline was 0.86 [95% confidence interval (CI) 0.57-1.15, z = 5.75, p < 0.00001]. Because of the small number of included RCTs, the preplanned sensitivity and subgroup analyses were finally abandoned. The dropouts in both groups were relatively low, indicating the high acceptability of rTMS.

**Conclusions:**

For TRD patients, augmentative rTMS after the failure of medications significantly increases the effect of antidepressants, and rTMS was a safe strategy with relatively low adverse events and low dropout rate, suggesting that augmentative rTMS is an effective intervention for TRD.

**Electronic supplementary material:**

The online version of this article (doi:10.1186/s12888-014-0342-4) contains supplementary material, which is available to authorized users.

## Background

Depression is a global severe disease. In a report of world health organization (WHO) [1], MDD ranked thirdly in global disease burden in 2004 and ranked firstly in moderate and high income countries. Unfortunately, even under the guidelines of the most competent specialists, more than 30% of patients cannot achieve a clinical response to an antidepressant regimen or psychotherapy [2]; these patients, which are called treatment-resistant depression (TRD) patients, exerted extremely severe burden on themselves and their families. So the treatment of TRDs has become one of the most important and pressing problem in psychiatry. With the development of neuroscience and psychiatry, we are now able to treat some TRDs with some physical method, such as electroconvulsive therapy (ECT), repetitive transcranial magnetic stimulation (rTMS) and transcranial direct current stimulation tDCS). Among these methods, ECT is the oldest and most effective while often criticized by the adverse effects of seizure induction and cognitive side effects [3,4], and patients who accepted ECT treatment often have to bear the stigma on this therapy [5].the limitations of ECT has resulted great interest in the two newly non-invasive neuromodulation methods: rTMS and tDCS, which are both of relatively low adverse effects and similar magnitude of antidepressant effects compared with antidepressant drugs [6].

rTMS utilizes an electromagnet that generates local magnetic field pulses to modulate brain functions. It is believed that rTMS modulates the activity of local neural circuits by decreasing or increasing the excitability of cortical neurons, depending on the parameters of stimulation. Usually, frequency higher than 5Hz is considered to increase cortical excitability and frequency lower than 1Hz decrease [7,8]. In view of this function, researchers have studied the efficacy of rTMS on different psychiatric disorders, such as schizophrenia and depression, based on the hypothesis of alleviating psychiatric symptoms by modulating local brain activity. In 1995 George et al. [9] firstly tried to apply rTMS in the treatment of MDD. After that, numerous literatures about the efficacy of rTMS on MDD have been published. In these studies, the parameters of rTMS were most commonly set as high frequency stimulation focused on the left dorsal lateral prefrontal cortex (LDLPFC) and low frequency on the right dorsal lateral prefrontal cortex (RDLPFC) [10].

At present, several meta-analyses [11-26] have indicated that rTMS is effective for MDD patients. But most studies had focused on both treatment-resistant and non-resistant patients and had chosen continuous data, namely, change from baseline of HAMD or MADRS scores as the primary outcome except for the study reported by Lam et al. [19], which focused on the treatment-resistant depression patients and chose dichotomous data, such as response rates and remission rates as the primary outcomes. The former kind of data tends to reserve the information of each RCT as much as possible, while the latter is easier for doctors and patients to understand. Nevertheless, the study by Lam et al. contained unexpected high heterogeneity, and included RCTs that designed rTMS as either monotherapy or augmentation to antidepressants, which may be on suspicion of mixing studies of great heterogeneity together.

In view of the above disadvantages of the previous meta-analyses, we designed this meta-analysis focused on RCTs which studied the efficacy of rTMS used as an augmentative strategy for antidepressants in treatment-resistant depression. The term “treatment-resistant” in this study is defined as failing to respond to at least one adequate antidepressant treatment [27], the “adequate treatment” means the medication should have been taken for at least 4 weeks and the following dose levels were considered adequate: for tricyclic antidepressants a dose equivalent to impramine 200 mg daily; for SSRIs a dose equivalent to paroxetine 40 mg daily; for venlafaxine 225 mg daily; and for mirtazapine 60 mg daily [28,29]. And “augmentative” in this study means either combination of rTMS and stable antidepressant treatment or simultaneous association of medication regimen and rTMS. Response rates and NNT were chosen as the primary outcomes. The baseline of HAMD scores, remission rates and dropout rates were selected as secondary outcomes. We synthesized the data of RCTs meeting the inclusion criteria in order to reduce the complexity and contradictions of different RCTs with relatively small samples collected from available databases and literatures. Sensitivity and subgroup analyses were conducted further to explore the source of heterogeneity and potential factors which may influence the efficacy of rTMS on TRD.

## Methods

### Search strategy

We identified articles for inclusion in this meta-analysis by:

1. Searching MEDLINE and Cochrane Central Register of Controlled Trials (CENTRAL) from 1 January 1995 to 30 November 2013, using the key words “transcranial magnetic stimulation”, “depress*”, “augment*”, “combin*”, “adjunctive”, “resistant” and ”refractory”. We restricted the article type to “randomized controlled trial” and the language to “English”.
2. Searching the references of all the previous relevant meta-analyses [11-26] focused on the efficacy of rTMS for MDD published earlier than 30 November 2013, as well as of all included RCTs.

The search procedures are described in details in Additional file 1.

### Inclusion criteria and exclusion criteria

Studies included in this meta-analysis should satisfy the following criteria:

1. Study validity: random allocation; double-blind (i.e. both patients and outcome raters were blind to the allocation); sham-controlled; rTMS was used as augmentation to antidepressants;
2. Sample characteristics: subjects should be 18–75 years old with a diagnosis of MDD according to DSM-IV or ICD-10, comorbidity of psychotic symptoms was excluded;
3. Efficacy evaluation and outcome reporting: efficacy should be rated by HAMD (17- or 21-items) or MADRS, and data are reported in a continuous (means and standard deviations (SDs) of pre- and post- treatment HAMD or MADRS scores) or dichotomous (response, remission and dropout rates) form able to be synthesized in this meta-analysis.
4. Articles should be published in English.

Studies were excluded if they were:

1. Non-RCT design, such as open trials;
2. Subjects were limited to a specific type of MDD patients, such as postpartum MDD, old MDD or secondary depression (i.e., vascular depression);
3. Sample size smaller than 5 in either rTMS or sham group;

### Data extraction

1. Sample characteristics: mean age, gender, diagnosis criteria and definition of treatment resistance;
2. rTMS-related: frequency, intensity, location, treatment strategy (number of sessions, duration of each stimulation and duration of each interval) and total pulses;
3. Drug-related: drug strategy (standardized or non-standardized), washout, types and dosages of each type;
4. Primary outcome measure: number of responders based on the RCTs’ primary efficacy measure (defined as ≥50% reduction in post-treatment on the HAMD or MADRS scores) at the end of blinded treatment;
5. Secondary outcome measure: number of remitters based on the RCTs’ primary efficacy measure (e.g. 17- or 21-item HAMD scores ≤7 or ≤8, respectively, or MADRS scores ≤10 [30]) at the end of blinded treatment, or the means and SDs of change of 17 or 21-item HAMD or MADRS scores;
6. Acceptability of treatment: number of dropouts in rTMS and sham groups in each RCT.

### Data synthesis and analysis

This meta-analysis was conducted according to the Cochrane Handbook for Systematic Reviews of Intervention [31], all statistic work were performed by Review Manager 5.2 and Excel 2007.

We used a random-effect model because the effcicacy of rTMS between different RCTs was assumed to be varied considerably. This model endows small-sample studies with higher weight and leads to a relatively conservative result [32]. For dichotomous data, if available, an intention-to treat analysis was selected. In other words, we included all dropouts after randomization, because this is closer to clinical practice. When dropouts were excluded for efficacy assessment in any individual RCT (e.g. subjects who never returned for assessment after randomization), they were considered as non-responders. We calculated the pooled OR of response rate and associated NNT. As reported in other studies, an NNT ≤ 10 was considered as clinically meaningful because such a treatment difference would be regularly encountered in clinical practice [33]. For continuous data, we calculated standardized mean difference (SMD) of the baseline HAMD or MADRS scores and change from baseline of HAMD or MADRS scores after the blinded treatment between active rTMS and sham groups. When a study had more than 2 groups, the data of different active rTMS groups were combined together as one group (the data were combined only when the active groups did not show significant difference, if they did, the RCT were excluded).

Heterogeneity was assessed by chi-square and I-square statistics [34], which is considered to be an indicator of study heterogeneity when p value for χ^2^ was lower than 0.1 or when I^2^ was higher than 35%. Sensitivity and subgroup analysis were conducted to determine the potential factors, such as sessions (≤10 or >10), intensity (≤100% or >100%) or total pulses (≤10000 or >10000), which may influence the antidepressant efficacy of rTMS. Nevertheless, because the number of included RCTs was relatively small, the heterogeneity of studies in subgroup analysis was considerably high, which lowered the reliability of the results. Finally, we used funnel plot and visual inspection to examine the publication bias.

## Results

### Literature search and screening

**Figure 1:**
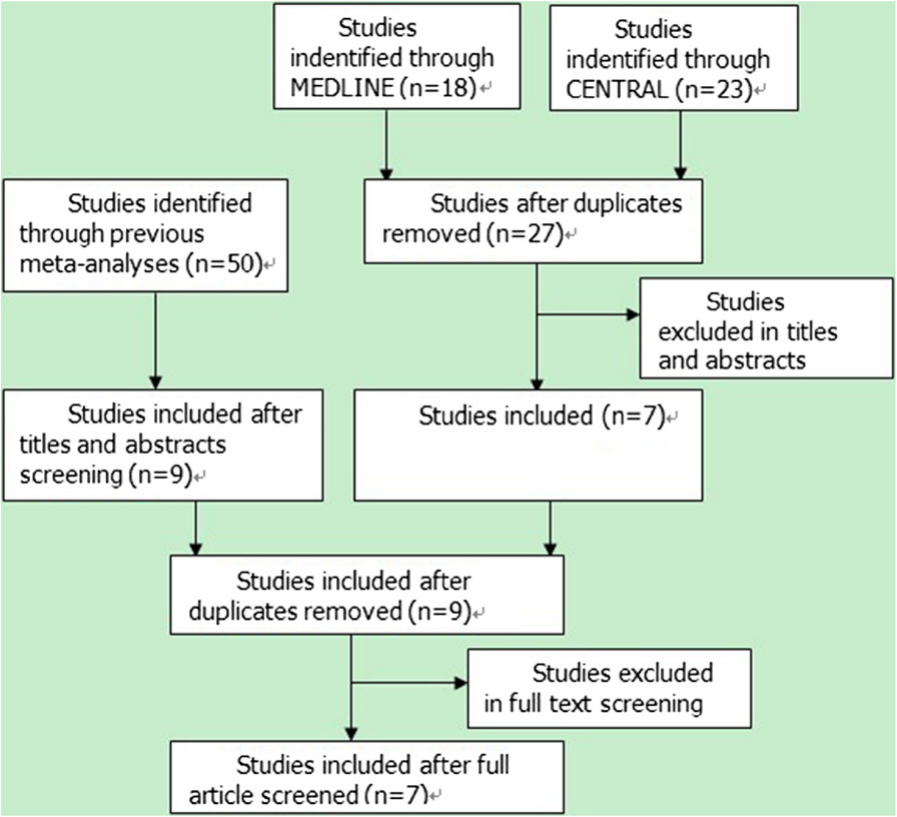
**Flowchart of literature search and screening.**

All the 7 RCTs [27,30,35-39] included in this meta-analysis were identified by electronic database searching, and the hand searching of bibliographies of previous meta-analyses did not result in additional studies available for data synthesis; two RCTs [40,41] on rTMS’s augmentative effect for TRD obtained by hand searching were excluded, because the data described in these two reports are unable to synthesize in this study. The process of literature search and screening was shown in Figure 1 and a detailed description of the process was available in Additional file 1.

### Included studies: main characteristics

**Figure 2:**
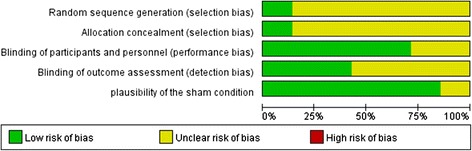
**Risk of bias graph: review authors' judgments about each risk of bias item presented as percentages across all included studies.**

All included RCTs [27,30,35-39] used HAMD as a primary outcome measurement. They all mentioned randomization, while no one described the scheme of allocation concealment in details. Three studies [30,38,39] reported how they guaranteed the blindness of the patients and the efficacy raters, while the other 4 RCTs [27,36,37] just mentioned “the patients and raters did not know which group they were allocated”. The risk of bias table was shown in Figure 2. All the studies had applied high frequency (≥5Hz) rTMS on the LDLPFC except for the study of Garcia-toro et al. [37], which simultaneously applied high frequency rTMS on the LDLPFC and low frequency rTMS on the RDLPFC. The main characteristics of included RCTs were described in Table 1.

**Table 1:** **Main characteristics of included RCTs**

**Study**	**Sample size (n)**		**Diagnosis**	**TRD definition**	**Medication regimen**	**rTMS parameters**				**Outcome assessment**	**Follow-up?**	**Adverse effects**
	**rTMS**	**sham**				**Frequency,Hz**	**Intensity (%MT)**	**Sessions**	**Total pulses**			
*Garcia-Toro et al.* [36]	17	18	DSM-IV MDD	Failed 2 or more ADs, 6 weeks minimum	continue current treatment	20	90	10	12000	HAMD-21	2w of an open trial	Scalp discomfort and slight headaches
*Rossini et al.* [38]	18	17	DSM-IV MDD & ≥ 26 in HAMD-21	Failed 2 or more ADs, 6 weeks minimum	continue current treatment	15	100	10	6000	HAMD-21	3w of follow up	Mild headache; discomfort at the site of stimulation
	19					15	80		6000			
*Garcia-Toro et al.* [37]	10	10	DSM-IV MDD	Failed 2 or more ADs, 1 month minimum	continue current treatment	20,1	110	10	12000	HAMD-21	2w of follow up	Scalp discomfort and headaches
	10					20,1	110		12000			
*Bretlau et al.* [27]	22	23	DSM-IV MDD	Failed 2 or more ADs, 6 weeks minimum	Escitalopram from 10 mg/d to 20 mg/d	8	90	15	19200	HAMD-17	9w of follow up	Reduced sleep length and greater concentration difficulties (sham group)
*Martinot et al.* [39]	16	14	DSM-IV-R MDD& ≥18 in MADRS & ≥16 in HAMD	Failed 2 or more ADs, 4 weeks minimum	minimal and stable dosage of previous treatment	10	90	10	16000	HAMD-21	N	Not mentioned
	18					10	90		16000			
*Bakim et al.* [30]	12	12	DSM-IV MDD & ≥18 in MADRS & ≥20 in HAMD	Failed 2 or more ADs, 6 weeks minimum	continue current treatment	20	80	30	24000	HAMD-17	N	Mild headache and mild discomfort
	11					20	100		24000			
*Chen et al.* [35]	10	11	DSM-IV MDD & ≥ 18 in HAMD-17	Failed 2 or more ADs, 6 weeks minimum	continue current treatment, stable dose	20	90	10	8000	HAMD-17	9w of follow up	Not mentioned(1 dropouts for unspecific somatic complaints, sham group)

### Response rates

**Figure 3:**
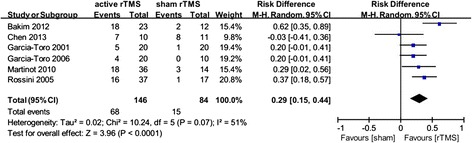
**Meta-analysis of active rTMS** ***vs*** **sham condition used as an augmentative strategy for antidepressants in treatment-resistant depression: response rates.**

Six RCTs had reported qualified data about response rates. As a whole, 68/146 (46.6%) and 15/84 (22.1%) subjects in the active or sham rTMS groups were classified as responders, respectively. The pooled OR was 5.12 (95% CI 2.11-12.45, z = 3.60, p = 0.0003), implying a significant difference favoring the active rTMS group (Figure 3). The risk difference translated into NNT was 3.4, namely, one patient would get clinical response in every 3.4 patients being treated.

**Figure 4:**
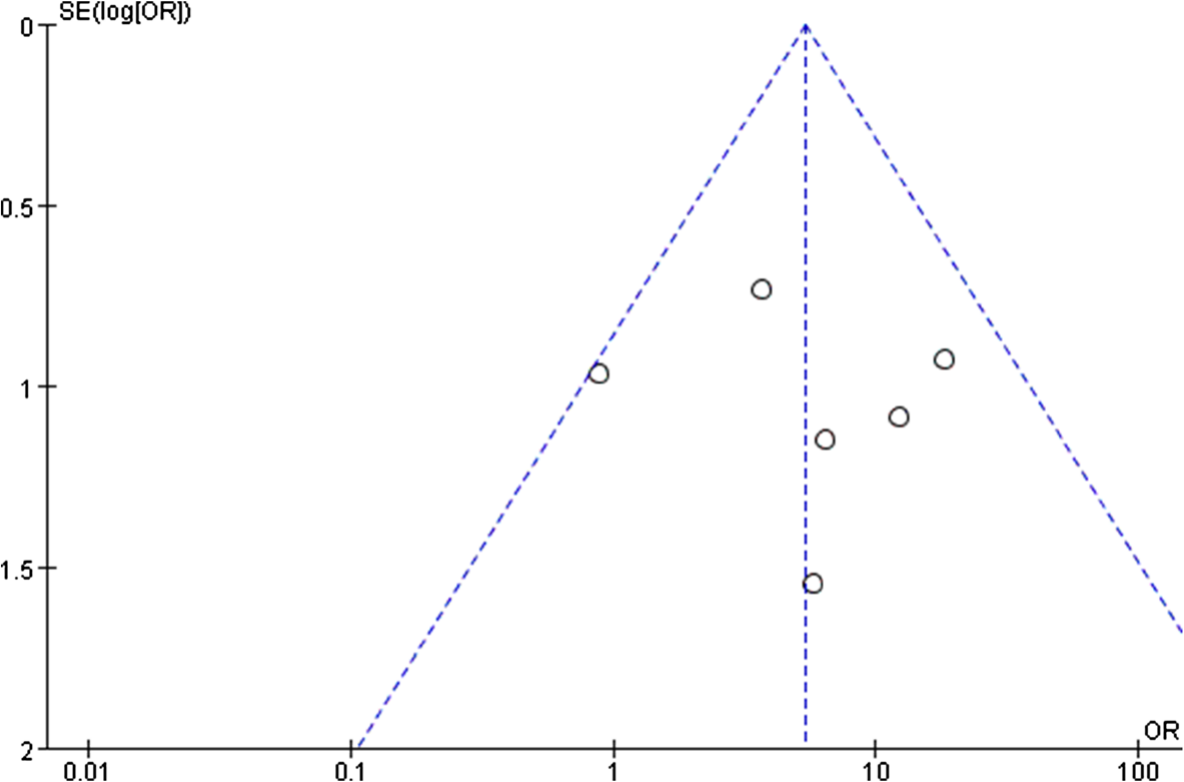
**Funnel plot of standard error by log odds ratio: response rates.**

Heterogeneity between RCTs did not exceed that expected by chance (χ^2^ = 6.09, p = 0.30, I^2^ = 18%), meaning that the variance among the effect sizes was no greater than that expected by sampling error. The associated Funnel Plot (Figure 4) is roughly symmetric, please refer to the Additional file 1.

### Change from baseline of HAMD scores

**Figure 5:**
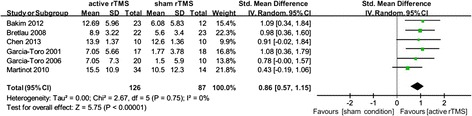
**Meta-analysis of active rTMS** ***versus*** **sham condition used as an augmentative strategy for antidepressants in treatment-resistant depression: change from baseline in HAMD scores.**

Data relating to change from baseline of HAMD scores were available from 6 RCTs, with a total of 126 and 87 subjects in the active rTMS and sham group, respectively. The pooled SMD was 0.86 (95% CI 0.57-1.15, z = 5.75, p < 0.00001), indicating the superiority of active rTMS in alleviating depression severity compared with sham rTMS (Figure 5).

Heterogeneity between RCTs did not exceed that expected by chance (χ^2^ = 2.67, p = 0.75, I^2^ = 0%), indicating that the data were reasonably appropriate for synthesis. The associated Funnel Plot was approximately symmetrical. For the associated funnel plot, please refer to the Additional file 1.

### Acceptability of treatment

All the 7 studies included in this meta-analysis reported the data of dropouts. In total, 9/171 (5.7%) and 5/108 (5.04%) dropped after the blinded treatment in the active rTMS and sham groups, respectively. The dropout rates in both groups were relatively low and had no significant difference [risk difference (RD) = 0.01, 95% CI −0.04-0.07, z = 0.48, p = 0.63] (because there were no dropouts in two RCTs [30,37], to enroll the data of the two studies, we chose RD rather than OR), indicating the relatively low adverse effect and high acceptability of rTMS. Additionally, the side effects reported by patients were presented in Table 1. The most frequent reported side effects were mild headache and discomfort in stimulation location, the headache and discomfort were commonly transient and did not differ significantly between groups, implying the safety of rTMS. For the associated forest plot and funnel plot, please refer to the Additional file 1.

### Remission rate

Only 2 RCTs [30,38] reported the number of remitters at the end of blinded rTMS treatment, both of them found a significant difference between the active and sham groups in achieving remission. For the small number or RCTs, data were not synthesized. For detailed description, please refer to the Additional file 1.

### rTMS *vs* sham group: baseline depression severity

No significant difference were observed in baseline severity between active and sham rTMS groups, the pooled SMD was −0.09 [95% CI −0.34-0.17, z = 0.66, p = 0.51], indicating that the two groups are comparable at baseline and baseline depression severity cannot be a confounding factor of the efficacy. For the associated forest plot and funnel plot, please refer to the Additional file 1.

### Sensitivity and subgroup analyses

We tried to carry out sensitivity and subgroup analyses to explore the potential confounding factors, such as number of sessions, intensity and total pulses, and drug strategy (standardized or non-standardized). However, because the number of included studies is relatively low and the heterogeneity between RCTs in subgroups were very high, the preplanned subgroup analyses were finally abandoned to avoid the misleading results. For detailed description, please refer to the Additional file 1.

### Follow-up data

Two studies [36,37] implemented open intervention after the blinded treatment. In these follow-up studies, patients in sham groups who did not achieve response in blinded treatment were enrolled to receive active rTMS treatment, both studies reported additional reduction of HAMD scores reduction after the “subsequent rTMS treatment”. The other three studies [27,35,38] reported non-interventional observation of the follow-up efficacy of rTMS., Interestingly, in the study of Chen et al. [35], augmentative active rTMS didn’t achieve significant difference compared to sham condition, while after 1 month’s follow-up, the active group showed significant greater HAMD scores reduction than the sham group, indicating a lagging efficacy of rTMS treatment, while in the study of Bretlau et al. [27], the results is inverse: rTMS group achieved significant greater HAMD scores reduction after treatment than the sham group, while after 9 weeks of follow-up, the difference is no longer significant between the groups. And the study of Rossini et al. [38] demonstrated a figure that shows active rTMS group achieved greater HAMD scores reduction both after treatment and in the follow-up duration than sham group, and the follow-up data in this study is not available.

## Discussion

To our knowledge, this is the first meta-analysis exploring the efficacy of augmentative rTMS for TRD. We included 7 RCTs and our results demonstrated that rTMS is significantly superior to sham condition in TRD treatment, the pooled OR was 5.12 (95% CI 2.11-12.45, z = 3.60, p = 0.0003), and the associated NNT was 3.4, indicating a relatively high efficacy of rTMS in TRD treatment. Moreover, our results showed that patients receiving rTMS treatment achieved significantly greater decrease in HAMD scores than those who receiving sham condition (SMD = 0.97, 95% CI 0.64-1.31, z = 6.00, p < 0.00001). Additionally, our results manifested that the baseline depression severity and dropout rates of the two groups did not differ significantly.

Although more than 10 meta-analyses about the efficacy of rTMS on depression had been published, there was only one meta-analysis [19] focused on the treatment-resistant patients. Nevertheless, the study by Lam et al. found a relatively high heterogeneity between the included RCTs, and this may be due to their enrollment of studies which used rTMS either as monotherapy or augmentation to antidepressants. Our results overcome the weakness by limiting the included studies only to the RCTs using rTMS as an augmentative strategy. For other meta-analyses, most of them had chosen change from baseline of HAMD scores or end-point HAMD scores as the primary outcome assessment, this may facilitate reserving the information of each individual study, but the outcomes of these studies are usually complicated and not easy for doctors and patients to understand. In this study, we provided an easily understood result by choosing response rate and NNT as the primary outcomes.

Despite the advantages aforementioned, our study has some limitations. Firstly, the strict inclusion criteria in this study may help to reduce heterogeneity and enhance the reliability of our results, but it may also limit our results only suitable for the condition of augmentative rTMS for TRD patients, thus we cannot know the efficacy of rTMS used as monotherapy for TRD patients or used as augmentative strategy for non-resistant MDD patients from the results of our study. However, that is not we intentioned to know and previous meta-analyses [14,19,24] had told us the answers to the above questions. Secondly, the quality of included RCTs is relatively low. Like other relevant meta-analyses [14,19], most included RCTs in this study did not report the method of allocation concealment and implementation and maintenance of blinding, which may lower the scoring of study quality. Thirdly, as mentioned in other meta-analyses and RCTs, the sham condition used in most studies cannot fully eliminate the placebo effect [10], because some patients receiving rTMS treatment can perceive the vibration of electromagnetic coil while the patients in the sham group cannot experience the effect. Moreover, the “5-cm” location method is often criticized for its inaccuracy [42,43], and it is unknown where is the exact stimulated location. Fourth, the follow-up duration of included studies was relatively low, and most studies designed open rTMS treatment in the follow up period. These factors make it impossible to estimate the median- or long-term naturalistic efficacy of rTMS. Fifth, as the number of included RCTs was relatively low, the predetermined subgroup analyses were finally not successfully conducted for the high heterogeneity. Therefore, it is unclear whether the intensity, frequency and total pulses of rTMS and drug strategy had contributed to the accuracy of the present results, and it should be settled by future studies.

## Conclusions

This study showed that augmentative rTMS was significantly superior to sham condition in TRD treatment, and the two groups did not differ significantly in dropout rates or side effects, indicating the advantage of rTMS in the efficacy and acceptability for clinical treatment of TRD. As the number of included RCTs was relatively low and the heterogeneity of RCTs in subgroup analysis is higher than expected by chance, we did not explore the potential confounding factors which may influence the effect of rTMS. Future studies should have more rigid design, such as reporting the method of allocation concealment and blinding in details, carrying out longer non-interventive follow-up to make clear of long-term efficacy of rTMS, and improving the design of sham condition to alleviate placebo effect further. Additionally, combination with neuroimaging technique for identifying the best stimulation location is expected.

## Additional file

Additional file 1:
**Detailed description of the process and results of this study.**
