# Supplementary material for: Repetitive transcranial magnetic stimulation as an augmentative strategy for treatment-resistant depression, a meta-analysis of randomized, double-blind and sham-controlled study
Source: BMC Psychiatry. 2014 Nov 30;14:342. doi: 10.1186/s12888-014-0342-4 (PMC4264336; doi:10.1186/s12888-014-0342-4)
Supplement: Additional file 1: — Detailed description of the process and results of this study. [file 12888_2014_342_MOESM1_ESM.doc]

**Supplementary material**

1.Literature search and screening

Electronic databases searching

(1)MEDLINE

search terms: (((((((TMS OR transcranial magnetic stimulation OR rTMS[Title]))) AND depress*[Title/Abstract]) AND (adjunctive OR augment* OR adjuvant OR combin*[Title/Abstract]))) AND (resistant OR refractory[Title/Abstract])) AND Randomized Controlled Trial[ptyp] AND English[lang]

results:18 references（1）

(2)CENTRAL

search terms:depress* in Title, Abstract, Keywords and TMS or rTMS or transcranial magnetic stimulation in Title, Abstract, Keywords and augment* or adjunctive or adjuvant or add-on or accelerat* or combin* in Title, Abstract, Keywords and resistant or refractory in Title, Abstract, Keywords in Trials'

results: 25 references with 2 duplicates, 23 references after discarding the duplicates（2）

（1）+（2）, imported into endnote ：41 references

Of the 41 references, 14 were duplicates, which were discarded after title screening. After the abstract screening, 20 studies were excluded for the following reasons:

1 for adolescents

1 for elderly

4 no full text

2 no sham condition

2 non drug-resistant

2 not adjunctive design

4 not for or not totally for MDD patients

3 not rTMS

1 with only patial patients being resistant

Finally, 7 studies obtained by electronic search and screening were included

Hand searching

The hand searching of references previous meta-analyses indentified 50 RCTs studying the efficacy of rTMS on depression, after the title and abstract screening, only 9 RCTs satisfied the inclusion criteria, and 7 of which were just the same as the articles indentified by electronic databases screening.

Later, the 9 articles were screened for full text, and 2 articles were further discarded in this process. Interestingly, the 2 studies discarded were both identified by hand search. **Fig 1** showed an explicit description of the literature searching and screening process:


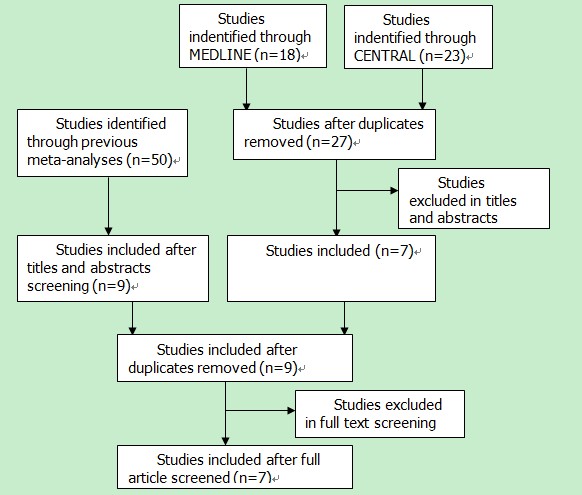


**Figure 1**: flowchart of literature search and screening.

Study evaluation

**
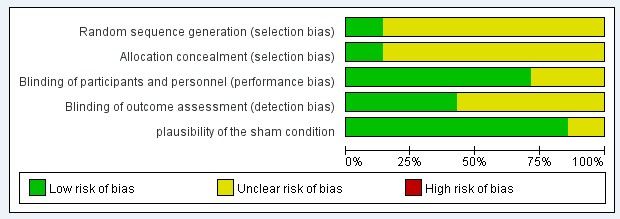
**

**Figure 2.** Risk of bias graph: review authors' judgements about each risk of bias item presented as percentages across all included studies.

2. Data synthesis and analysis


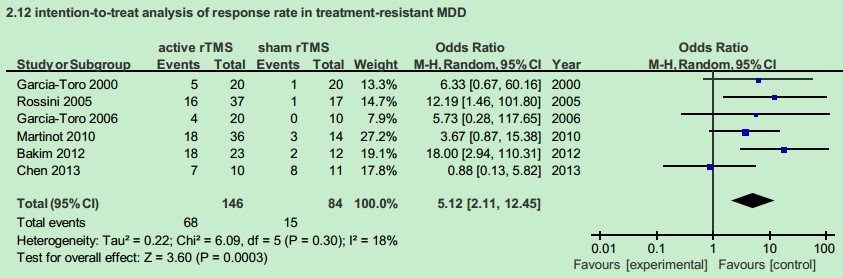
Response rates:

**Figure 3**. Meta-analysis of active rTMS *vs* sham condition used as an augmentative strategy for antidepressants in treatment-resistant depression: response rates


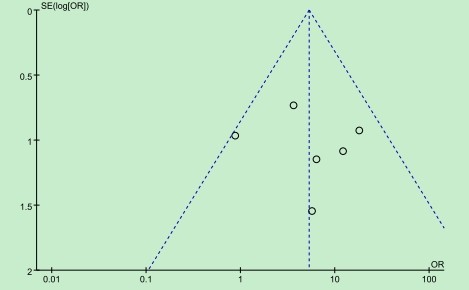


**Figure 4.** Funnel plot of standard error by log odds ratio: response rates. The funnel plot is roughly symmetric.

Change from baseline of HAMD scores


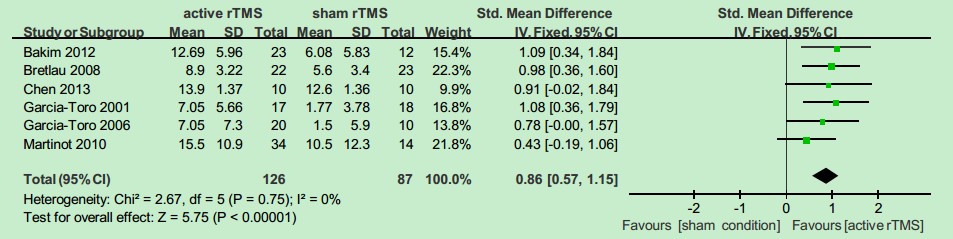


**Figure 5.** Meta-analysis of active rTMS *versus* sham condition used as an augmentative strategy for antidepressants in treatment-resistant depression: change from baseline in HAMD scores.


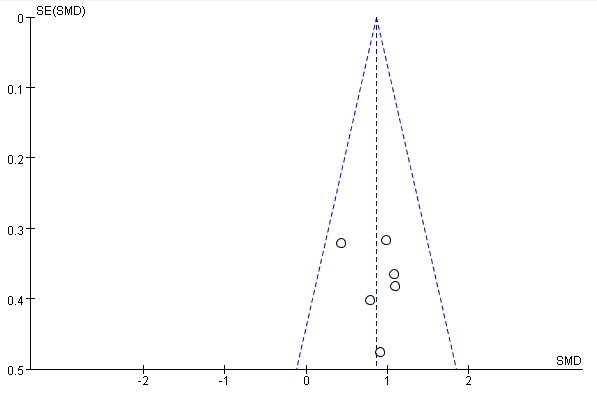


**Figure 6.** Funnel plot of standard error by log odds ratio: change from baseline of HAMD scores. The funnel plot is roughly symmetric.

Acceptability (Dropout rates)


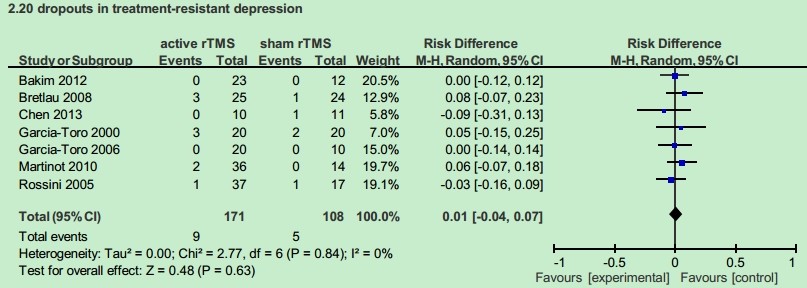


**Figure 7.** Meta-analysis of active rTMS *versus* sham condition used as an augmentative strategy for antidepressants in treatment-resistant depression: dropout rates.


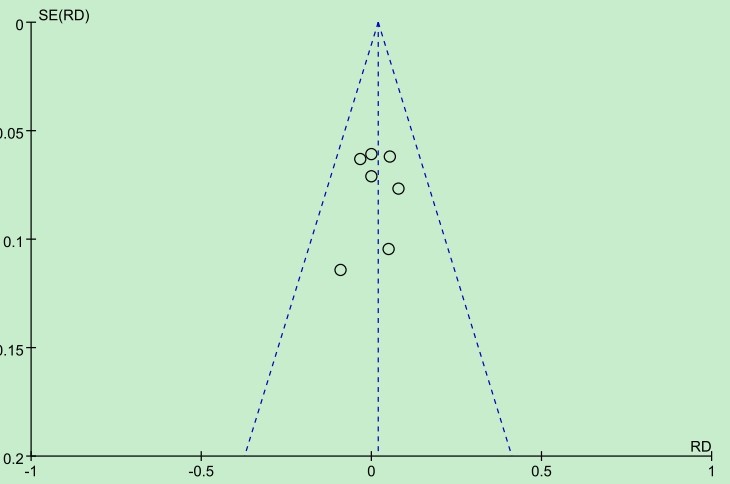


**Figure 8.** Funnel plot of standard error by log odds ratio: dropout rates. The funnel plot is roughly symmetric.

Remission rates:

Only 2 RCTs reported the number of remitters at the end of blinded rTMS treatment. In the study reported by Bakim et al 2012, the remission rates of the active rTMS and sham groups were 27.3 (9/33) and 8.3% (1/12), respectively. In the study reported by Rossini et al 2005, the remission rates of the active rTMS and sham groups were 37.8% (14/37) and 0% (0/17), respectively. Both of the studies found a significant difference between the two groups in remission rate.

Baseline HAMD scores:


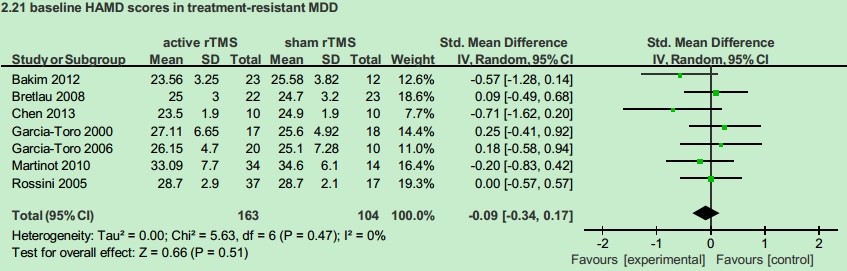


**Figure 9.** Meta-analysis of active rTMS *versus* sham condition used as an augmentative strategy for antidepressants in treatment-resistant depression: baseline HAMD scores.


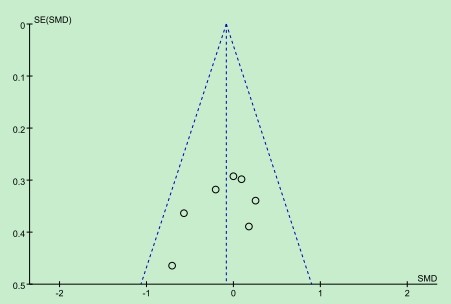


**Figure 10.** Funnel plot of standard error by log odds ratio: baseline HAMD scores.

Sensitivity analysis：

Sessions : studies with sessions≤10. Forest plot and funnel plot:


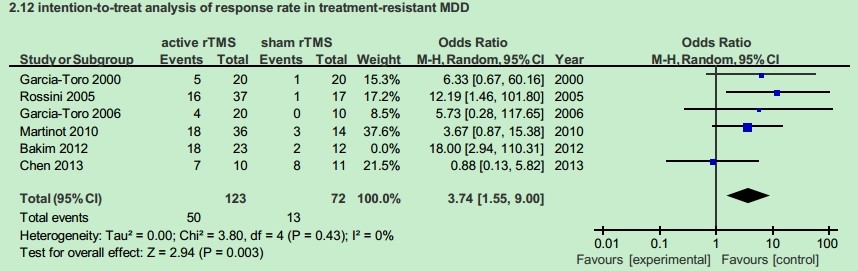


Although the heterogeneity is still low in subgroup analysis, we can’t infer from the results that the number of sessions exerted influence on the efficacy, because there is only one study with sessions>10, and there is no linear correlation between the OR and number of sessions, which can be roughly seen from the above forest plot.

Intensity: MT%

Studies with the intensity of rTMS ≤100%. Forest plot and funnel plot:


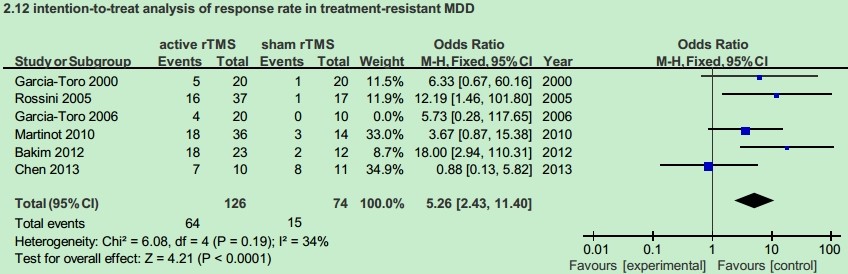


We can see that the heterogeneity was increased after the study of Garcia-Toro 2006 removed.

Total pulses：total pulses≥10000


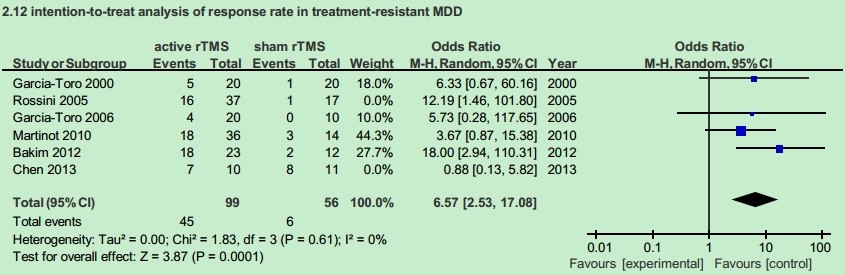


Like the sessions, there is no linear correlation between the ORs and total pulses.

Because all included RCTs chose high frequency rTMS, and the frequencies used are all relatively high, so it is not easy to divide the RCTs in to subgroups according to frequency

1. Wall CA, Croarkin PE, Sim LA, Husain MM, Janicak PG, Kozel FA, Emslie GJ, Dowd SM, Sampson SM: **Adjunctive use of repetitive transcranial magnetic stimulation in depressed adolescents: A prospective, open pilot study**. In: *Journal of Clinical Psychiatry.* vol. 72; 2011: 1263-1269.

2. Mosimann UP, Schmitt W, Greenberg BD, Kosel M, Muri RM, Berkhoff M, Hess CW, Fisch HU, Schlaepfer TE: **Repetitive transcranial magnetic stimulation: a putative add-on treatment for major depression in elderly patients**. *Psychiatry Res* 2004, **126**(2):123-133.

3. Bretlau LG, Lunde M: **Treatment of therapy-resistant major depression with transcranial stimulation (TMS) in combination with escitalopram [NCT00149071]**. In: *ClinicalTrialsgov [wwwclinicaltrialsgov].* 2006.

4. Dell'Osso B, Castellano F, Camuri G, Arici C, Altamura AC: **Efficacy and tolerability of different stimulation parameters of augmentative repetitive transcranial magnetic stimulation (rTMS) in the acute treatment of drug-resistant depression [conference abstract]**. In: *Biological Psychiatry [abstracts from the 66th Annual Meeting of the Society of Biological Psychiatry San Francisco, CA United States May 12-14 2011].* vol. 69; 2011.

5. Loo CK, Mitchell PB, McFarquhar TF, Malhi GS, Sachdev PS: **A sham-controlled trial of the efficacy and safety of twice-daily rTMS in major depression**. In: *Psychological medicine.* vol. 37; 2007: 341-349.

6. Zhang XH, Wang LW, Wang JJ, Liu Q, Fan Y: **Adjunctive treatment with transcranial magnetic stimulation in treatment resistant depression: a randomized, double-blind, sham controlled study**. In: *Shanghai Archives of Psychiatry.* vol. 23; 2001: 17-24.

7. Eche J, Mondino M, Haesebaert F, Saoud M, Poulet E, Brunelin J: **Low-vs high-frequency repetitive transcranial magnetic stimulation as an add-on treatment for refractory depression**. In: *Frontiers in psychiatry.* vol. 3; 2012: ArtID 13.

8. Fitzgerald PB, Huntsman S, Gunewardene R, Kulkarni J, Daskalakis ZJ: **A randomized trial of low-frequency right-prefrontal-cortex transcranial magnetic stimulation as augmentation in treatment-resistant major depression**. In: *The international journal of neuropsychopharmacology / official scientific journal of the Collegium Internationale Neuropsychopharmacologicum (CINP).* vol. 9; 2006: 655-666.

9. Poulet E, Brunelin J, Boeuve C, Lerond J, D'Amato T, Dalery J, Saoud M: **Repetitive transcranial magnetic stimulation does not potentiate antidepressant treatment**. In: *European psychiatry : the journal of the Association of European Psychiatrists.* vol. 19; 2004: 382-383.

10. Rossini D, Magri L, Lucca A, Giordani S, Smeraldi E, Zanardi R: **Does rTMS hasten the response to escitalopram, sertraline, or venlafaxine in patients with major depressive disorder? A double-blind, randomized, sham-controlled trial**. In: *The Journal of clinical psychiatry.* vol. 66; 2005: 1569-1575.

11. Fitzgerald PB, Benitez J, Castella A, Daskalakis ZJ, Brown TL, Kulkarni J: **A randomized, controlled trial of sequential bilateral repetitive transcranial magnetic stimulation for treatment-resistant depression**. In: *The American journal of psychiatry.* vol. 163; 2006: 88-94.

12. McDonald WM, Easley K, Byrd EH, Holtzheimer P, Tuohy S, Woodard JL, Beyer K, Epstein CM: **Combination rapid transcranial magnetic stimulation in treatment refractory depression**. In: *Neuropsychiatric Disease and Treatment.* vol. 2; 2006: 85-94.

13. Kang JI, Kim CH, Namkoong K, Lee CI, Kim SJ: **A randomized controlled study of sequentially applied repetitive transcranial magnetic stimulation in obsessive-compulsive disorder**. In: *The Journal of clinical psychiatry.* vol. 70; 2009: 1645-1651.

14. Mogg A, Purvis R, Eranti S, Contell F, Taylor JP, Nicholson T, Brown RG, McLoughlin DM: **Repetitive transcranial magnetic stimulation for negative symptoms of schizophrenia: a randomized controlled pilot study**. In: *Schizophrenia research.* vol. 93; 2007: 221-228.

15. Picarelli H, Teixeira MJ, Andrade DC, Myczkowski ML, Luvisotto TB, Yeng LT, Fonoff ET, Pridmore S, Marcolin MA: **Repetitive transcranial magnetic stimulation is efficacious as an add-on to pharmacological therapy in complex regional pain syndrome (CRPS) type I**. In: *The journal of pain : official journal of the American Pain Society.* vol. 11; 2010: 1203-1210.

16. Su TP, Huang CC, Wei IH: **Add-on rTMS for medication-resistant depression: a randomized, double-blind, sham-controlled trial in Chinese patients**. In: *The Journal of clinical psychiatry.* vol. 66; 2005: 930-937.

17. Palm U, Schiller C, Fintescu Z, Obermeier M, Keeser D, Reisinger E, Pogarell O, Nitsche MA, Moller HJ, Padberg F: **Transcranial direct current stimulation in treatment resistant depression: a randomized double-blind, placebo-controlled study**. *Brain stimulation* 2012, **5**(3):242-251.

18. Martiny K, Lunde M, Bech P: **Transcranial low voltage pulsed electromagnetic fields in patients with treatment-resistant depression**. In: *Biological psychiatry.* vol. 68; 2010: 163-169.

19. Sobis J, Jarzab M, Hese RT, Sieron A, Zyss T, Gorczyca P, Gierlotka Z, Pudlo R, Matysiakiewicz J: **Therapeutic efficacy assessment of weak variable magnetic fields with low value of induction in patients with drug-resistant depression**. *J Affect Disord* 2010, **123**(1-3):321-326.

20. Anderson IM, Delvai NA, Ashim B, Ashim S, Lewin C, Singh V, Sturman D, Strickland PL: **Adjunctive fast repetitive transcranial magnetic stimulation in depression**. In: *The British journal of psychiatry : the journal of mental science.* vol. 190; 2007: 533-534.

21. Aguirre I, Carretero B, Ibarra O, Kuhalainen J, Martinez J, Ferrer A, Salva J, Roca M, Gili M, Montoya P *et al*: **Age predicts low-frequency transcranial magnetic stimulation efficacy in major depression**. *J Affect Disord* 2011, **130**(3):466-469.

22. Pallanti S, Bernardi S, Di Rollo A, Antonini S, Quercioli L: **Unilateral low frequency versus sequential bilateral repetitive transcranial magnetic stimulation: is simpler better for treatment of resistant depression?** *Neuroscience* 2010, **167**(2):323-328.

23. Bakim B, Uzun UE, Karamustafalioglu O, Ozcelik B, Alpak G, Tankaya O, Cengiz Y, Yavuz BG: **The combination of antidepressant drug therapy and high-frequency repetitive transcranial magnetic stimulation in medication-resistant depression**. *BCP* 2012, **22**(3):244-253.

24. Rossini D, Lucca A, Zanardi R, Magri L, Smeraldi E: **Transcranial magnetic stimulation in treatment-resistant depressed patients: a double-blind, placebo-controlled trial**. In: *Psychiatry research.* vol. 137; 2005: 1-10.
